# Non-coding RNA-mediated high expression of *SFXN3* as a prognostic biomarker associated with paclitaxel resistance and immunosuppressive microenvironment in head and neck cancer

**DOI:** 10.3389/fimmu.2022.920136

**Published:** 2022-09-08

**Authors:** Kailin Chen, Sha Gong, Xueliang Fang, Qian Li, Mingliang Ye, Junyan Li, Shengyan Huang, Yuheng Zhao, Na Liu, Yingqin Li, Jun Ma

**Affiliations:** ^1^ Department of Radiation Oncology, State Key Laboratory of Oncology in South China, Collaborative Innovation Center of Cancer Medicine, Guangdong Key Laboratory of Nasopharyngeal Carcinoma Diagnosis and Therapy, Sun Yat-sen University Cancer Center, Guangzhou, China; ^2^ Department of Experimental Research, State Key Laboratory of Oncology in South China, Collaborative Innovation Center of Cancer Medicine, Guangdong Key Laboratory of Nasopharyngeal Carcinoma Diagnosis and Therapy, Sun Yat-sen University Cancer Center, Guangzhou, China

**Keywords:** sideroflexin 3, paclitaxel resistance, immune infiltration, targeted therapy, head and neck cancer

## Abstract

Chemoresistance is the leading cause of poor prognosis in head and neck squamous cell carcinoma (HNSC); however, promising biomarkers to identify patients for stratified chemotherapy are lacking. Sideroflexin 3 (*SFXN3*) is an important mitochondrial serine transporter during one-carbon metabolism, which is involved in the proliferation of cancer cells. However, the specific role of *SFXN3* in HNSC remains unknown. In this study, we performed expression and survival analysis for *SFXN3* in pan-cancer using data from The Cancer Genome Atlas (TCGA) and Genotype-Tissue Expression (GTEx) and found that *SFXN3* served as a potential oncogene in HNSC. Notably, *SFXN3* expression was found to be positively associated with enriched tumor-infiltrating macrophages, other immune suppressive cells, and immune checkpoint expression and resistance to paclitaxel. Gene, clinical, and immune variables included in the univariate and multivariate analyses showed that *SFXN3* expression was an independent risk factor. Moreover, the *LINC01270*/hsa-miR-29c-3p/*SFXN3* axis was identified as the most likely upstream non-coding RNA-related pathway of *SFXN3* in HNSC using bioinformatic analysis, expression analysis, correlation analysis, and survival analysis. Taken together, our findings demonstrated that a non-coding RNA-mediated high expression of *SFXN3* is a prognostic biomarker and is associated with the immunosuppressive microenvironment in HNSC.

## Introduction

Head and neck cancer is the seventh most common cancer globally, and squamous cell carcinoma is the most common histological type (1, 2). The majority of patients (>50%) with head and neck squamous cell carcinoma (HNSC) present with a locally advanced stage or distant metastasis at diagnosis (3). Despite multimodal therapeutic interventions, the outcome of patients with advanced-stage HNSC remains unsatisfactory: 40%–60% of these patients ultimately experience recurrence or local progression, with poor prognosis (4). Therapeutic resistance, especially chemoresistance, is the leading cause of poor prognosis (2, 5). Therefore, identifying promising biomarkers for stratified chemotherapy and prognosis, and novel therapeutic targets for the effective management of HNSC, are urgently required.

Sideroflexin 3 (*SFXN3*) is a member of the sideroflexin family, of which there are five homologs of these mitochondrial proteins in humans. The expression of *SFXN* homologs varies across tissues (6). The *SFXN3* protein level is relatively high in the mature brain and neurons (7–9). Consistent with these finding, *SFXN3* was reported to be involved in neurodegenerative diseases, including Parkinson’s disease, Alzheimer’s disease, and other neurological dysfunction diseases (10–12). It is widely accepted that many cancer cells are highly dependent on serine metabolism for their growth and proliferation (10, 13, 14). *SFXN3*, one of the main mitochondrial serine transporters during one-carbon metabolism, might play a crucial role in the development of cancer. However, few studies have focused on the role of *SFXN3* in cancer. Murase et al. reported that serum autoantibodies to *SFXN3* might be a novel tumor marker for oral squamous cell carcinoma (15). These findings prompted us to conduct a comprehensive study of the expression, prognosis, and possible mechanism of *SFXN3* in HNSC. Moreover, although *SFXN3* was reported to be expressed in the stromal fibroblasts around cancer nests (15), the correlation of *SFXN3* with drug resistance and tumor immune infiltration in HNSC has not been determined.

In this study, we analyzed the expression and prognostic value of *SFXN3* in pan-cancer. Next, we determined the effect of *SFXN3* expression on drug sensitivity. Importantly, the roles of *SFXN3* in HNSC immune infiltration were further explored. Mechanistically, non-coding RNAs (ncRNAs) related to the regulation of *SFXN3*, such as long non-coding RNAs (lncRNAs) and microRNAs (miRNAs), were also investigated in HNSC. Taken together, our findings suggested that the ncRNA-mediated upregulation of *SFXN3* was associated with poor prognosis, paclitaxel resistance, and the immunosuppressive microenvironment in HNSC.

## Materials and methods

### TCGA data preprocessing

The normalized pan-cancer dataset, TCGA Pan-Cancer (PANCAN, N = 10,535, G = 60,499), was downloaded from the UCSC (https://xenabrowser.net/) database. The samples of common cancer types were used for further analysis, including glioblastoma multiforme (GBM), cervical squamous cell carcinoma and endocervical adenocarcinoma (CESC), lung adenocarcinoma (LUAD), colon adenocarcinoma (COAD), breast invasive carcinoma (BRCA), esophageal carcinoma (ESCA), stomach and esophageal carcinoma (STES), kidney renal papillary cell carcinoma (KIRP), stomach adenocarcinoma (STAD), prostate adenocarcinoma (PRAD), uterine corpus endometrial carcinoma (UCEC), head and neck squamous cell carcinoma (HNSC), kidney renal clear cell carcinoma (KIRC), lung squamous cell carcinoma (LUSC), liver hepatocellular carcinoma (LIHC), thyroid carcinoma (THCA), rectum adenocarcinoma (READ), pancreatic adenocarcinoma (PAAD), pheochromocytoma and paraganglioma (PCPG), bladder urothelial carcinoma (BLCA), kidney chromophobe (KICH), and cholangiocarcinoma (CHOL). Then, we extracted the expression data of the ENSG00000107819 (*SFXN3*) gene in each sample with log2(x+0.001) transformation for each expression value.

In addition, transcriptional profiles of count values and clinicopathological characteristics for HNSC (n = 546) were also obtained from the UCSC (https://xenabrowser.net/) database. Normalization was conducted by the “limma” package. The univariate and multivariate Cox regression analyses for survival in HNSC and CIBERSORTx and xCell analysis were conducted.

### Differential expression and survival analysis

The Wilcoxon rank-sum test was used to compare expression values between normal and tumor samples in pan-cancer analysis (16). Cox regression was used to analyze the relationship between gene expression and prognosis in each tumor. The log-rank test was used to compare two survival curves. The Sangerbox online tool (17) was used for statistical analysis and graphing. The UALCAN and Gene Expression Profiling Interactive Analysis (GEPIA) databases were used to further confirm these results. UALCAN (http://ualcan.path.uab.edu/index.html) is a web resource providing a comprehensive analysis of cancer transcriptome data, such as that from TCGA (18). Transcripts per million (TPM) values were employed to estimate the significance of the differences in gene expression levels between groups using a *t* test. GEPIA (http://gepia.cancer-pku.cn/) is a web tool for analyzing RNA sequencing expression and correlation data of cancer and normal samples from TCGA and the Genotype-Tissue Expression (GTEx) data (19). *SFXN3* and lncRNA expressions on box plots were determined by GEPIA in various types of human cancer. The method used for differential analysis was one-way ANOVA, and the *p* value cutoff was 0.05. Survival analysis for *SFXN3* expression and candidate lncRNAs in cancers was also performed at GEPIA using the log-rank test. The statistical analyses of the online databases in this study were calculated automatically. Median values were employed as group cutoffs, and a log-rank *p* value < 0.05 was identified as statistically significant.

### Tumor immune estimation resource, CIBERSORTx, and xCell analysis

Tumor Immune Estimation Resource (TIMER) is a comprehensive resource for the analysis of tumor-infiltrating immune cells across more than 30 types of cancer (https://cistrome.shinyapps.io/timer/) (20). In the present study, we compared tumor infiltration levels among tumors with different somatic copy number alterations for *SFXN3* and the correlation of *SFXN3* expression with tumor immune infiltration using TIMER in the HNSC dataset.

Moreover, we applied the CIBERSORTx computational method to calculate the proportion of 22 subtypes of immune cells in case of HNSC from TCGA database (21). According to the cell type description in the LM22 signature matrix, 11 major leukocyte types were divided into 22 leukocyte subsets (22). The classification of immune cells was unified based on the description of comparative data from other databases more conveniently. We analyzed the fractions of the 10 main types of immune cells, including B cells (naïve B cells and memory B cells), plasma cells, CD8 T cells, CD4 T cells (CD4 naïve T cells, CD4 memory resting T cells, CD4 memory activated T cells, T follicular helper cells (Tfhs), and regulatory T cells (Tregs)), gamma delta T cells, natural killer (NK) cells (resting NK cells, activated NK cells), monocytes, macrophages (M0 macrophages, M1 macrophages, M2 macrophages), dendritic cells (resting dendritic cells, activated dendritic cells), and neutrophils. xCell (https://xcell.ucsf.edu/) is another signature-based method for quantifying the infiltration of immune cells in tissues. We used the “xCell” R package to generate immune estimates to validate the CIBERSORT results by R software (version 3.6.4) (23).

### Assessment of the prognostic role of *SFXN3* in relation with the immune infiltration

We employed a univariate Cox regression to identify the variables with a close relationship to OS in HNSC patients. The variables in the analysis included *SFXN3* expression, the levels of immune cell infiltration by CIBERSORT, clinical data (age, T stage, N stage, and stage), and tumor purity. The high and low groups of immune cell infiltration levels were determined based on the median values. Tumor purity estimates on HNSC were obtained from a previous study (24). Four methods were used for tumor purity estimates: ESTIMATE, ABSOLUTE, LUMP, and IHC. ESTIMATE applied gene expression profiles of 141 immune genes and 141 stromal genes to evaluate (25). Somatic copy-number data were used for ABSOLUTE (26). LUMP is the abbreviation of leukocytes unmethylation for purity; it averages 44 non-methylated immune-specific CpG sites. IHC was estimated by image analysis of hematoxylin and eosin stain slides (24).

All covariates with a *p* value less than 0.1 based on univariate analysis were included in the multivariate model. The backward Wald method was used for the multivariate Cox proportional hazards regression model by SPSS 24.0 software (IBM, Armonk, NY, USA).

### Drug sensitivity analysis

To clarify the association between *SFXN3* expression and drug sensitivity and tolerance, we downloaded transcriptome data and drug sensitivity half-maximal inhibitory concentration (IC50) values of the NCI-60 panel of human cancer cell lines from the CellMiner database (https://discover.nci.nih.gov/cellminer/) (27). The therapeutic effects of 574 kinds of drugs or compounds in clinical trials and 218 US FDA-approved drugs in the database were selected for further analysis. The effect of *SFXN3* expression on the drug sensitivity was analyzed using the “impute” (28) and “limma” R packages (29). Missing data for some drugs were imputed using impute. knn from the impute R package. There are 1,275 missing values, 5% of the total. Spearman’s correlation test was used to analyze the relationship between the activity of drugs or compounds and the expression of *SFXN3*. Linear models were used for analysis of drug sensitivity. The Wilcoxon test was utilized to analyze the difference in the activity of drugs between the high- and low-risk *SFXN3* expression groups, and then a box plot was drawn using the “ggplot2” and “ggpubr” functions of R.

To assess the relationship between *SFXN3* expression and the sensitivity of commonly used chemotherapy drugs for HNSC, we downloaded the HNSC cell line half-maximal inhibitory concentration (IC50) values of commonly used chemotherapy drugs from the Genomics of Drug Sensitivity in Cancer (GDSC) database (30). These values can be linked with the *SFXN3* expressions of the corresponding cell lines reported in the Cancer Cell Line Encyclopedia (https://sites.broadinstitute.org/ccle/). In the analysis of each drug, the expression of *SFNX3* was grouped high and low according to the median value. The Wilcoxon rank-sum test was used to compare IC50 values between the high and low *SFXN3* expression groups using the Sangerbox online tool.

### Prediction of candidate miRNAs and lncRNAs

The upstream miRNAs of *SFXN3* were predicted using eight target gene prediction programs, including DIANA TOOLS (31), miRDB (32), miRmap (33), miWalk (34), RNA22 (35), TargetScan (36), microT-CDS (37), and miRTarBASE (38). Only the miRNAs predicted in more than two programs were selected for subsequent analysis. CancerMIRNome (http://bioinfo.jialab-ucr.org/CancerMIRNome/) is a comprehensive database for the interactive analysis of miRNA expression profiles from TCGA projects (39). The datasets of significant downregulated miRNAs and positive prognostic miRNAs in HNSC were downloaded from CancerMIRNome. Pearson’s correlation analysis was conducted between the expression of hsa-miR-29c-3p and the expression of *SFXN3*.

The upstream candidate lncRNAs of hsa-miR-29c-3p were predicted using the ENCORI database (https://starbase.sysu.edu.cn/index.php) (40). Correlation analyses for lncRNA–hsa-miR-29c-3p or lncRNA–*SFXN3* and the expression level of lncRNAs in HNSC were performed at ENCORI using Pearson’s correlation coefficient.

## Results

### Pan-cancer analysis of *SFXN3* expression and its prognostic value

**Figure 1:**
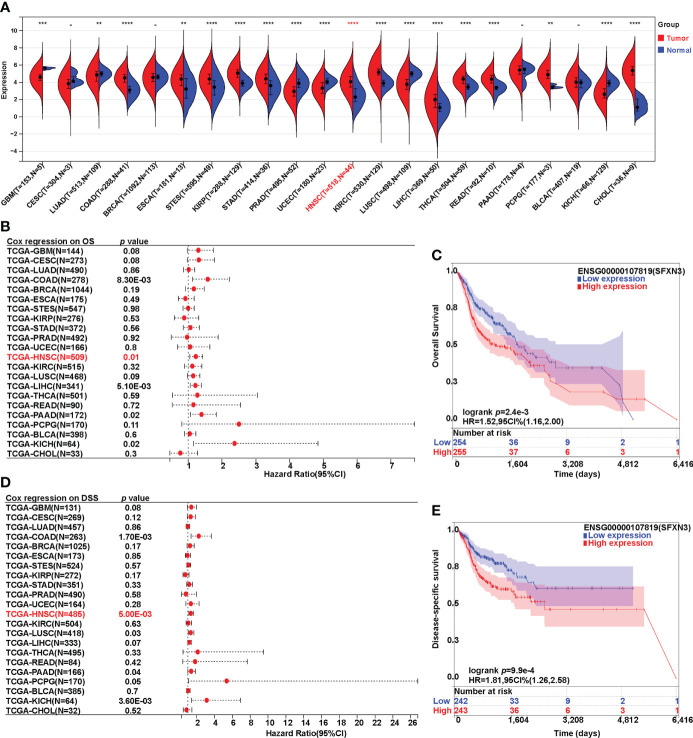
Expression analysis and survival analysis for *SFXN3* in pan-cancer. **(A)** The expression of *SFXN3* in pan-cancer based on TCGA cancer and normal data analyzed. **(B)** Cox regression analysis on overall survival was performed on *SFXN3* expression in pan-cancer. **(C)** The overall survival (OS) analysis in patients with HNSC. **(D)** Cox regression analysis on disease-specific survival (DSS) was performed on *SFXN3* expression in pan-cancer. **(E)** The DSS analysis for *SFXN3* expression in patients with HNSC determined. -: no significant difference, **p value < 0.01; ***p value < 0.001; ****p value < 0.0001.

To explore the possible roles of *SFXN3* in pan-cancer, we first analyzed its expression in 22 common cancer types. Compared with that in the normal samples, the expression of *SFXN3* was significantly upregulated in 12 cancer types, namely, COAD, ESCA, STES, KIRP, STAD, HNSC, KIRC, LIHC, THCA, READ, PCPG, and CHOL (all *p <* 0.01, **Figure 1A**), while the expression of *SFXN3* was significantly downregulated in GBM, LUAD, PRAD, UCEC, LUSC, and KICH. Next, the UALACAN and GEPIA databases were used to validate the expression of *SFXN3* in pan-cancer. As shown in **Figure S1A**, the expression of *SFXN3* was significantly upregulated in five cancer types by the UALACAN database, including CHOL, COAD, HNSC, KIRC, KIRP, LIHC, READ, THCA, and STAD (all *p* < 0.001). *SFXN3* expression levels in CHOL, HNSC, KIRC, acute myeloid leukemia (LAML), PAAD, PCPG, and sarcoma (SARC) were upregulated compared with those in the corresponding normal controls in the GEPIA database (all *p* < 0.05, **Figure S1B**). Taken together, these data demonstrated that *SFXN3* was upregulated in CHOL, HNSC, and KIRC, indicating that *SFXN3* might act as a crucial regulator in the carcinogenesis of these three cancer types.

Furthermore, Cox survival analysis was performed on *SFXN3* expression in pan-cancer. Both overall survival (OS) and disease-specific survival (DSS) analyses were conducted. For OS, a higher expression of *SFXN3* in patients was associated with significantly worse prognosis in COAD, HNSC, LIHC, PAAD, and KICH (*p* = 0.008, 0.01, 0.005, 0.02, and 0.02, respectively). COAD, HNSC, and KICH were consistent with a high expression in the tumor (**Figures 1B, C**). However, patients with ESCA, STES, KIRP, STAD, KIRC, THCA, READ, PCPG, and CHOL with a higher expression of *SFXN3* showed no significance in terms of OS (all *p* > 0.05).

For DSS, Cox regression analysis found that a higher expression of *SFXN3* demonstrated poorer prognosis in COAD and HNSC (*p* = 0.002 and 0.005, respectively, **Figures 1D, E**). In addition, survival analysis was also performed in GEPIA, including CESC, CHOL, HNSC, KICH, KIRC, LUSC, PAAD, PCPG, PRAD, and UCEC. A higher expression of *SFXN3* in patients was associated with significantly worse prognosis in HNSC for overall survival (*p* = 0.006) (**Figure S1C**). For DFS, a higher *SFXN3* expression demonstrated poorer prognosis in HNSC (*p* = 0.037, **Figure S1D**).

These results demonstrated that *SFXN3* expression may serve as a negative and unfavorable prognostic marker for patients with HNSC.

### Poor prognosis of patients with HNSC with a high *SFXN3* expression was attributed to enriched tumor-infiltrating macrophages

**Figure 2:**
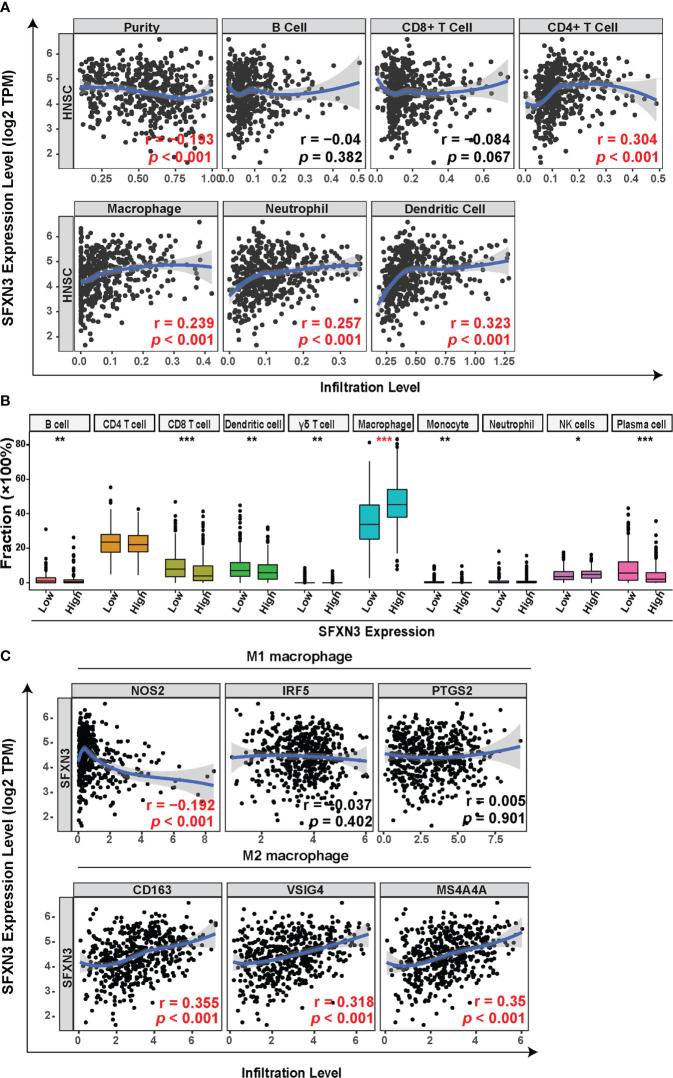
Correlation between *SFXN3* expression with tumor immune cell infiltration in patients with head and neck squamous cell carcinoma (HNSC). **(A)** Tumor Immune Estimation Resource (TIMER) was used to analyze the correlation between *SFXN3* expression and immune effector cells. **(B)** Tumor-infiltrating immune cells in HNSC samples were estimated using the CIBERSORT algorithm. **(C)** Correlation analysis of *SFXN3* expression with the markers of M1 or M2 polarized phenotypes of macrophages. *p value < 0.05; **p value < 0.01; ***p value < 0.001.

Head and neck cancer is a profoundly immunosuppressive disease with high immune infiltration (41, 42). Therefore, we evaluated the relationship between *SFXN3* expression and the level of immune infiltration in HNSC. As shown in **Figure 2A**, *SFXN3* expression was significantly and positively associated with the infiltration level of CD4^+^ T cells (r = 0.304, *p* < 0.001), macrophages (r = 0.239, *p* < 0.001), neutrophils (r = 0.257, *p* < 0.001), and dendritic cells (r = 0.323, *p* < 0.001), but not B cells (r = −0.04, *p* = 0.382) and CD8^+^ T cells (r = −0.084, *p* = 0.067). The CIBERSORT algorithm was used to further confirm these results. The levels of tumor‐infiltrating B cells, CD8^+^ T cells, dendritic cells, gamma delta T cells, macrophages, NK cells, and plasma cells correlated significantly with *SFXN3* expression (all *p* < 0.05, **Figure 2B**). Moreover, we found that the levels of tumor-infiltrating dendritic cells, macrophages, myocytes, and plasma cells also correlated significantly with *SFXN3* expression, as calculated using xCell (all *p* < 0.05, **Figure S2**)

Taken together, a high *SFXN3* expression was associated with significantly higher proportions of tumor-infiltrating macrophages. Correlation analyses between *SFXN3* expression and biomarkers of M1 or M2 polarized phenotypes of macrophages were further investigated. *SFXN3* expression correlated significantly and positively with biomarkers of immunosuppressive M2 macrophages (CD163: r = 0.355, *p* < 0.001; the V-set and immunoglobulin domain-containing protein 4 (VSIG4): r = 0.318, *p* < 0.001; and membrane-spanning 4-domains subfamily A 4A (MS4A4A): r = 0.35, *p* < 0.001) and correlated negatively with one of the M1 macrophages’ biomarkers (NO synthase 2 (NOS2): r = −0.192, *p* < 0.001) (**Figure 2C**).

The data demonstrated that a high expression of *SFXN3* in HNSC associated with tumor progression and poor prognosis might partly be related to enriched tumor-infiltrating macrophages.

### *SFXN3* expression is significantly related to the immunosuppressive microenvironment in HNSC

**Figure 3:**
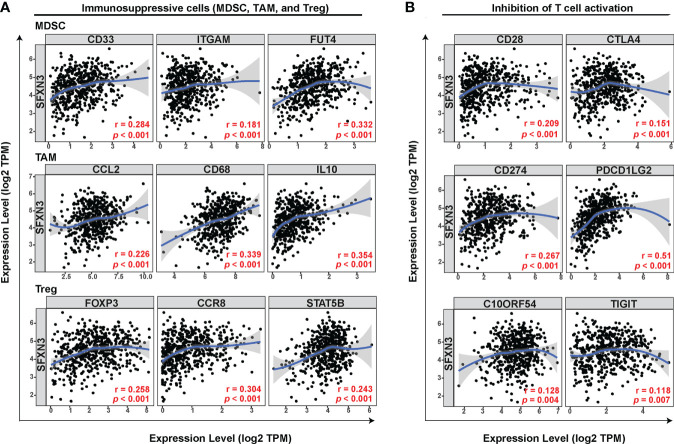
*SFXN3* expression correlates significantly with markers of immunosuppressive molecules and cells. **(A)** Correlation between *SFXN3* expression and markers of immunosuppressive cells (MDSCs, TAMs, and Tregs). **(B)** Correlation between *SFXN3* expression with molecules involved in inhibition of T-cell activation (*CD28*, *CTLA4*, *CD274*, *PDCD1LG2*, *VSI*R, and *TIGIT*).

In addition to tumor-associated macrophages (TAMs), especially M2 macrophages, myeloid-derived suppressor cells (MDSCs) and Tregs also contribute to forming an immunosuppressive microenvironment in tumor tissues (43). Next, we explored the relationship between *SFXN3* expression and biomarkers of the main immune-suppressive cells including MDSCs, TAMs, and Tregs in the tumor microenvironment. We conducted a correlation analysis using TIMER, which showed that the immune markers of MDSCs (CD33: r = 0.284, *p* < 0.001; integrin subunit alpha M (ITGAM): r = 0.181, *p* < 0.001, fucosyltransferase 4 (FUT4): r = 0.332, *p* < 0.001), TAMs (C–C motif chemokine ligand 2 (CCL2): r = 0.226, *p* < 0.001, CD68: r = 0.339, *p* < 0.001, interleukin (IL)-10: r = 0.354, *p* < 0.001), and Tregs (forkhead box P3 (FOXP3): r = 0. 258, *p* < 0.001, C–C motif chemokine receptor 8 (CCR8): r = 0.304, *p* < 0.001, signal transducer and activator of transcription 5 (STAT5B): r = 0.243, *p* < 0.001) were significantly and positively associated with the *SFXN3* expression level **(** **Figure 3A**) (44, 45). Inhibition of T-cell activation is one of the common causes contributing to macrophage-related tumor immune evasion (46). We further performed a correlation analysis between *SFXN3* expression and inhibitory factors involved in the T-cell activation (46). The analysis showed that the *SFXN3* expression level correlated positively with the expression of genes related to the inhibition of T-cell activation, including *CD28* (encoding CD28 molecule) (r =0.209, *p* < 0.001), *CTLA4* [encoding cytotoxic T lymphocyte-associated antigen-4) (r = 0.151, *p* < 0.001), *PDL1* (encoding programmed cell death 1 ligand 1 (also known as CD274)] (r = 0.267, *p* < 0.001), *PDCD1LG2* (encoding programmed cell death 1 ligand 2 (PD-L2)) (r = 0.51, *p* < 0.001), *VSIR* (encoding V-set immunoregulatory receptor (also known as C10ORF54); r = 0.128, *p* = 0.004), and TIGIT (encoding T-cell immunoglobulin and ITIM domain) (r = 0.118, *p* = 0.007) (**Figure 3B**).

These data indicated that a high *SFXN3* expression was associated with an immunosuppressive microenvironment, possibly *via* inhibition of T-cell activity.

### *SFXN3* expression was an independent risk factor

**Figure 4:**
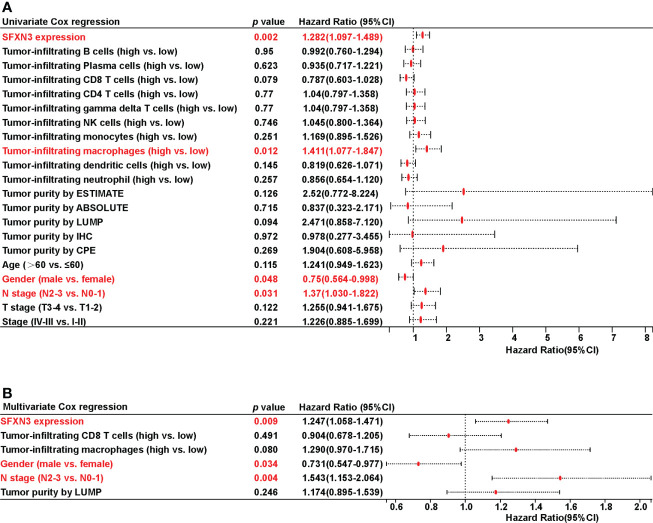
Univariate and multivariate Cox regression analyses on survival in patients with head and neck squamous cell carcinoma (HNSC). **(A)** Univariate Cox regression analysis on survival in HNSC. **(B)** Multivariate Cox regression analysis on survival in HNSC.

Univariate Cox regression analysis revealed that SFNX3 expression [hazard ratio (HR): 1.282 (1.097-1.489), *p* = 0.002], tumor-infiltrating macrophages [HR: 1.411 (1.077-1.847), *p* = 0.012], gender [HR: 0.75 (0.564-0.998), *p* = 0.048], and N stage [HR: 1.37 (1.030-1.822), *p* = 0.031] were significant predictors of OS in patients with HNSC, which are shown in **Figure 4A**.

All covariates with a *p* value less than 0.10 based on univariate analysis were included in the multivariate model. Only the following variables remained significant after multivariate adjustment: *SFXN3* [HR: 1.247 (1.058-1.471), *p* = 0.009], gender [HR: 0.731 (0.547-0.977), *p* = 0.034], and N stage [HR: 1.543 (1.153-2.064), *p* = 0.004], which are demonstrated in **Figure 4B**.

### The values of *SFXN3* in drug resistance analysis

**Figure 5:**
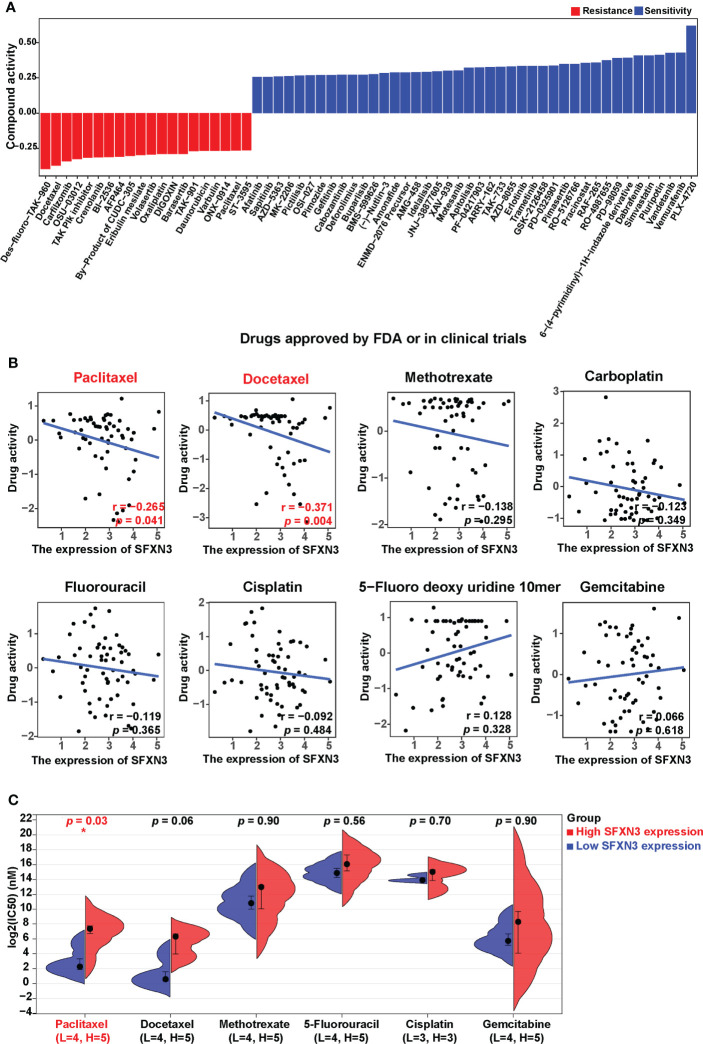
*SFXN3* expression as a potential predictor for drug sensitivity. **(A)** The compound activity z scores of chosen compounds in relation to *SFXN3* expression, as shown by correlation analysis. **(B)** Correlation analysis using the Spearman correlation test for *SFXN3* expression and activity z scores of paclitaxel, docetaxel, paclitaxel, methotrexate, carboplatin, fluorouracil, cisplatin, 5-fluoro deoxy uridine 10mer, and gemcitabine. **(C)** IC50 values of chemotherapeutics, including paclitaxel, docetaxel, methotrexate, 5-fluorouracil, cisplatin, and gemcitabine in the high *SFXN3* expression HNSC cell lines compared with those in the low *SFXN3* expression HNSC cell lines. HNSC cell lines with high *SFXN3* expression were found to possess higher IC50 for docetaxel. The *p*-values were calculated using the Wilcoxon rank-sum test. *p value < 0.05.

To investigate the potential values of *SFXN3* expression to predict drug resistance in cancer, we acquired NCI-60 compound activity and corresponding NCI-60 cell line RNA-seq/composite expression from the CellMiner database. Compound activity scores and sensitivity to the corresponding compound correlated positively. We further selected 792 drugs or compounds in the database that are closely related to clinical treatment, including 574 drugs in clinical trials and 218 US FDA-approved drugs, for further analysis. Overall, *SFXN3* expression was found to be associated with the increased activity of 42 drugs or compounds and the decreased activity of 20 drugs or compounds (**Figure 5A**).

According to the National Comprehensive Cancer Network (NCCN) guidelines, cisplatin, gemcitabine, paclitaxel, docetaxel, carboplatin, 5-FU, and methotrexate are commonly used chemotherapy drugs for head and neck cancer. Correlation analysis for these drugs showed that the activity of paclitaxel and docetaxel was negatively and significantly related to the expression of *SFXN3* (r = −0.265, *p* = 0.041, and r = −0.371, *p* = 0.004, respectively; **Figure 5B**). We also found a trend of negative correlation between *SFXN3* expression and the activity of cisplatin, carboplatin, or methotrexate, but without a significant difference (all *p* > 0.05). We also divided patients into high or low *SFXN3* expression groups according to the median value of *SFXN3* expression and then investigated whether *SFXN3* expression has an impact on the activity of the drugs. Patients with a high expression of *SFXN3* were found to possess a lower drug activity for paclitaxel and docetaxel (both *p* < 0.05, **Figure S3**).

In order to evaluate whether *SFXN3* expression has an impact on the activity of the drugs in HNSC, we divided the expression values of SFNX3 in different HNSC cell lines into two high and low groups, which are shown in supplementary material **Table S1**. HNSC cell lines with a high expression of *SFXN3* were confirmed to have higher IC50 values for paclitaxel (*p* = 0.03, **Figure 5C**). We also found a trend of the correlation between *SFXN3* expression and IC50 of docetaxel, but without a significant difference (*p* = 0.06). Moreover, there was no significant difference between *SFXN3* expression and the IC50 values of methotrexate, 5-FU, cisplatin, and gemcitabine (*p =*0.90, 0.56, 0.70, and 0.90, respectively).

These findings suggested that *SFXN3* expression might function as a paclitaxel resistance predictor, which might be closely related to poor prognosis in head and neck cancer.

### Prediction regulatory miRNAs of *SFXN3*

Non-coding RNAs can act as regulators of gene expression, which has been widely investigated. MicroRNAs (miRNAs) are short RNAs that perform their function by binding to the target mRNAs (47).

**Figure 6:**
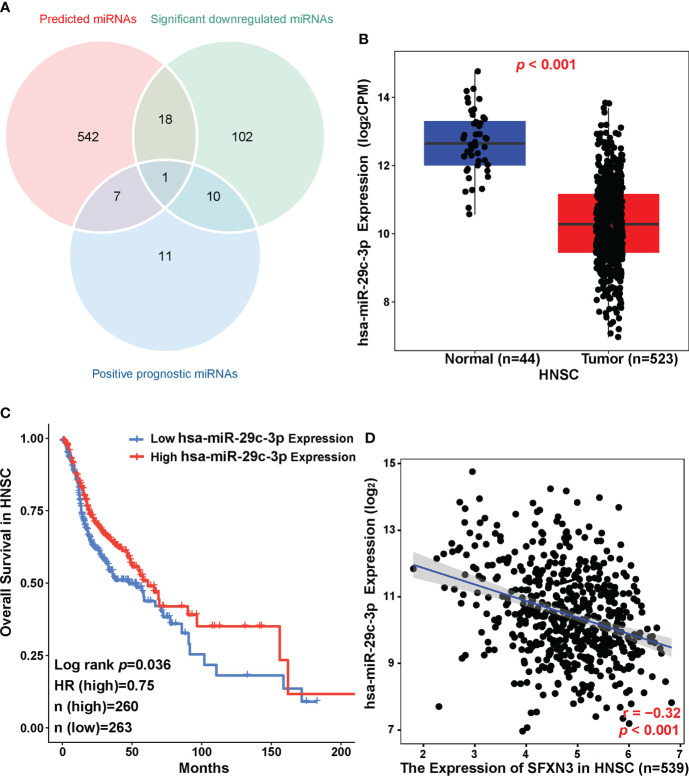
Identification of hsa-miR-29c-3p as a potential upstream miRNA of *SFXN3* in HNSC. **(A)** Venn diagram of predicted miRNAs, downregulated miRNAs, and miRNAs related to better survival in HNSC. **(B)** The expression of hsa-miR-29c-3p in HNSC and control normal samples as determined using the CancerMIRNome database. **(C)** The prognostic value of hsa‐miR‐29c‐3p in HNSC assessed using the CancerMIRNome. **(D)** The expression correlation between hsa-miR-29c-3p and *SFXN3* in HNSC.

To clarify the possible mechanism by which ncRNAs might mediate the regulation of *SFXN3*, we first predicted miRNAs that could bind to *SFXN3*. Using eight prediction programs, 568 predicted miRNAs were identified, which are shown in supplementary material **Table S2**. According to the mechanisms of the miRNA–target interaction, the predicted upstream miRNAs should correlate negatively with *SFXN3* expression. In the CancerMIRNome analysis of TCGA database, 29 positive prognostic miRNAs and 131 significantly downregulated miRNAs were found in HNSC (**Figure 6A**). Only hsa-miR-29c-3p met both these requirements. hsa-miR-29c-3p was significantly downregulated in HNSC, and its upregulation was associated with better prognosis of patients with HNSC (**Figures 6B, C**, *p* < 0.05). Pearson’s correlation analysis showed that *SFXN3* expression was also significantly and negatively correlated with hsa-miR-29c-3p expression (r = -0.32, *p* < 0.001) (**Figure 6D**). Taken together, hsa-miR-29c-3p might be an upstream miRNA of *SFXN3* in HNSC.

### Prediction of upstream lncRNAs of hsa-miR-29c-3p

**Figure 7:**
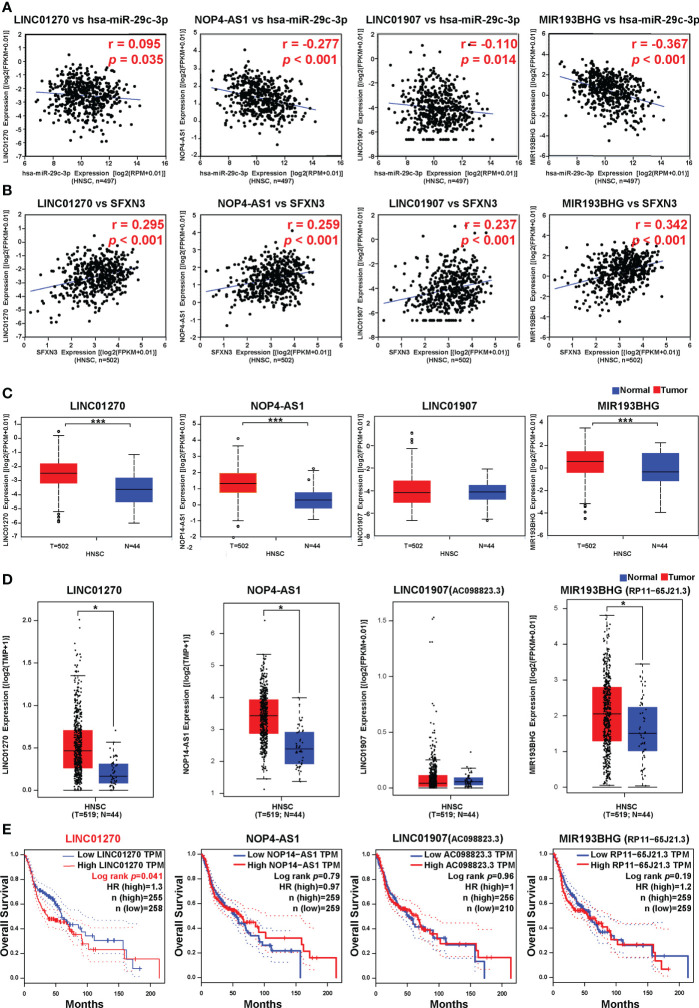
Correlation analysis, expression analysis, and survival analysis for upstream lncRNAs of hsa-miR-29c-3p in HNSC. **(A)** Correlation analysis between hsa-miR-29c-3p and *LINC01270*, *NOP14-AS1*, *LINC01907*, and *MIR193BHG*, respectively. **(B)** Correlation analysis between *SFXN3* and *LINC01270*, *NOP14-AS1*, *LINC01907*, and *MIR193BHG*. **(C)** The expression of *LINC01270*, *NOP14-AS1*, *LINC01907*, and *MIR193BHG* in TCGA HNSC compared with that in normal tissues using ENCORI. **(D)** The expression of *LINC01270*, *NOP14-AS1*, *LINC01907*, and *MIR193BHG* in TCGA HCC compared with “TCGA normal” or “TCGA and GTEx normal” data. **(E)** The OS analysis for *LINC01270*, *NOP14-AS1*, *LINC01907*, and *MIR193BHG* in HNSC. *p value < 0.05; ***p value < 0.01.

The upstream lncRNAs of hsa-miR-29c-3p were further predicted using the ENCORI database. A total of 114 possible lncRNAs were predicted. Among them, six predicted lncRNAs were negatively associated with the expression of hsa-miR-29c-3p in HNSC, including *AC098828.2* (ENSG00000223734), *LINC01270* (ENSG00000203999), *MIR193BHG* (ENSG00000262454), *PVT1* (ENSG00000249859), *NOP14-AS1* (ENSG00000249673), and *LINC0190*7 (ENSG00000226125). However, only four lncRNAs were positively associated with the expression of *SFXN3* (**Figures 7A, B**), which suggested that the lncRNAs were associated with a competing endogenous RNA (ceRNA) mechanism. Then, the expression levels of these four related lncRNAs in HNSC were assessed using both ENCORI and the GEPIA database. As suggested in **Figures 7C, D**, *LINC01270*, *NOP14-AS1*, and *MIR193BHG* were significantly upregulated in HNSC compared with that in normal controls and were consistent in the two databases. Subsequently, the prognostic values of these four lncRNAs were evaluated in HNSC. As shown in **Figure 7E**, patients with HNSCs with a higher expression of *LINC01270* were significantly related to poorer OS (*p* = 0.041).

Based on the results of the correlation, expression, and survival analyses, *LINC01270* was considered to be the most likely upstream lncRNA of the hsa-miR-29c-3p/*SFXN3* axis in HNSC.

## Discussion

In this study, the expression analysis and survival analysis for *SFXN3* indicated that HNSC patients with a high expression of *SFXN3* had poor prognosis. Correlation analysis and comparative analysis between groups based on *SFXN3* expression indicated that *SFXN3* might function as a paclitaxel and docetaxel resistance predictor and is related to enriched tumor‐infiltrating macrophages, immune-suppressive cells, and immune checkpoint expression.

*SFXN1* and *SFXN3* are the main mitochondrial serine transporters in human cells. Both of them are likely to be regulated by the Myc transcription factor, which is a well-known oncogene. Serine transported to mitochondria is converted into glycine and formate, while generating reactive one-carbon units. Glycine can also provide one-carbon units to the folate cycle (10). Although *SFXN3* is the closest homolog of *SFXN1*, loss of *SFXN3* causes a more severe defect in glycine synthesis defect compared with loss of *SFXN1*. Besides, *SFXN1* has no prognostic value for HNSC (**Figure S4**). Moreover, *SFXN3*, in addition to serine, can also transport alanine or cysteine. Cysteine is another product of serine metabolism. The two products of serine metabolism, glycine and cysteine, are precursors for glutathione synthesis. Moreover, serine and glycine are essential for tumor growth and survival (48). Metabolic requirements evolve during cancer progression, and new dependencies emerged in the context of therapy resistance and metastasis (49).

Both paclitaxel and docetaxel are microtubule-stabilizing agents, which are constituents of a classical chemotherapy regimen according to the guide for the treatment of HNSC (2). Recently, resistance to docetaxel in non-small cell lung cancer cells was reported to be closely related to serine and glycine metabolism and cysteine metabolism. More serine was found to be accumulated in the docetaxel-resistant non-small cell lung cancer cells compared with the levels in the sensitive cells (50). However, the abundance of enzymes involved in glutathione biosynthesis in docetaxel-resistant cells was dramatically lower than that in sensitive cells, which ultimately reduced the glutathione levels and elevated the levels of reactive oxygen species. Increased oxidative stress stimulated the expression and function of permeability glycoproteins and thus promoted drug resistance (50). Furthermore, serine and glycine metabolism was found to be increased in the more radioresistant HNSC cancer cells (51). These observations might indicate that HNSC cancer cells that are more resistant to therapy might have altered the metabolic pathways that control redox status, DNA repair, and DNA methylation after radiation (49). In our study, we found that *SFXN3* expression correlated with paclitaxel resistance by the analysis with data from head and neck tumor cell lines, and there was also a trend toward correlation with docetaxel resistance. Further investigations are needed to clarify whether resistance to paclitaxel and docetaxel in HNSC is associated with serine and glycine metabolism.

The standard clinical practices for HNSC have changed substantially in the era of immunotherapy. HNSC is a malignant tumor in which the immune surveillance mechanism is suppressed (41). Our study suggested that *SFXN3* expression correlated significantly and positively with biomarkers of the main immune-suppressive cells including MDSCs, TAMs, and Tregs, in the tumor microenvironment. In oral squamous cell carcinoma, *SFXN3* is not only expressed in cancer cells but also expressed in components of the tumor microenvironment, such as the stromal fibroblasts and the endothelial cells of the small arteries in the cancer nest (15). Increased levels of serine and glycine in tumor tissue were demonstrated to promote the survival of tumor stromal cells and vascular epithelial cells, which can establish a protective niche for the maintenance of the tumor (48). This may be one of the reasons why *SFXN3* is related to the tumor microenvironment. Second, the inflammatory response in macrophages is supported by serine metabolism. Inflammatory cytokine IL-1β, produced by macrophages, is associated with tumorigenesis in HNSC (52). Rodriguez et al. showed that glycine, which is made from serine, could support glutathione synthesis in macrophages, leading to the production of IL-1β. Furthermore, serine also supplies glycine and one-carbon units to support T-cell proliferation (53). However, there is a competitive relationship between tumor cells and T cells, and perhaps macrophages, for cysteine and glycine, which might be related to the suppression of T-cell activation and proliferation, leading to an immunosuppressive microenvironment (54).

Extensive research has shown that ncRNAs, including miRNAs, lncRNAs, and circular RNAs (circRNAs), can act as regulators of gene expression. Many ncRNAs function as competitive endogenous RNAs (ceRNAs) to regulate the biological behaviors of cancer (47, 55). To clarify whether a ceRNA regulatory mechanism existed in the regulation of *SFXN3*, we employed prediction programs to predict possible miRNAs that could potentially bind to *SFXN3* and possible related lncRNAs. The *LINC01270*/hsa-miR-29c-3p/*SFXN3* axis was identified as a potential regulatory pathway in HNSC. *LINC01270* was upregulated and was significantly associated with worse OS in HNSC, which was consistent with the role of *LINC01270* in lung adenocarcinoma, endometriosis, esophageal cancer, and breast cancer (56–59). Correspondingly, hsa-miR-29c-3p was downregulated and was significantly associated with better survival in HNSC in the present study, which was consistent with previous research in HNSC, bladder cancer, and colorectal cancer (60–63).

Our study had several limitations. First, the heterogeneity of head and neck cancer varies. The subsites of head and neck tumors and treatment modalities might have affected the identification of significant prognostic variables. Second, the potential upstream axis of *SFXN3* and the estimation of infiltrated immune cell types were computationally predicted. Experimental exploration and multicenter and prospective studies are needed to further verify the clinical value of *SFXN3* and its underlying mechanisms.

## Conclusion

**Figure 8:**
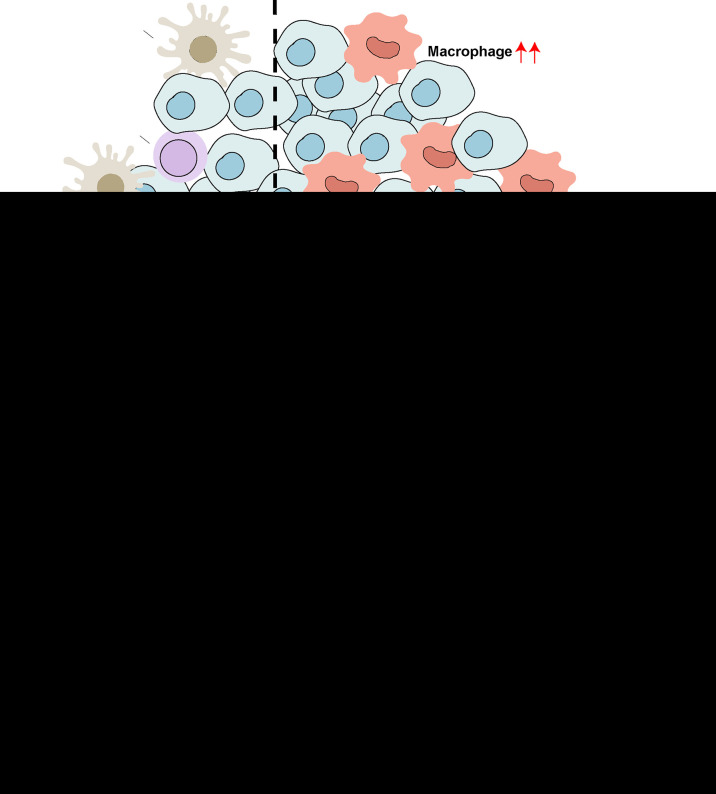
Schematic model of the *LINC01270*/hsa-miR-29c-3p/*SFXN3* axis as a potential regulatory pathway in HNSC.

We demonstrated that *SFXN3* was highly expressed and correlated with unfavorable prognosis in HNSC. We identified the *LINC01270*/hsa-miR-29c-3p/*SFXN3* axis as a potential regulatory pathway in HNSC (**Figure 8**). Furthermore, *SFXN3* might exert its oncogenic roles by increasing resistance to paclitaxel and promoting the immunosuppressive microenvironment in HNSC. However, further basic research and clinical trials are needed to validate these findings.

## Data availability statement

The R codes are accessible on Jianguoyun at: https://www.jianguoyun.com/p/DTLfw3QQkdi8ChiJsbkEIAA.

## Author contributions

KC and YL designed the article. KC, SG and QL organized the public data and wrote the manuscript. KC, XF, JL and YL took charge for data visualization. MY, SH and YZ obtained the clinical information. NL and JM contributed to the concept and revised the article. All authors contributed to the article and approved the submitted version.

## Funding

This study was supported by grants from the Natural Science Foundation of Guangdong Province (2017A030312003).

## Conflict of interest

The authors declare that the research was conducted in the absence of any commercial or financial relationships that could be construed as a potential conflict of interest.

## Publisher’s note

All claims expressed in this article are solely those of the authors and do not necessarily represent those of their affiliated organizations, or those of the publisher, the editors and the reviewers. Any product that may be evaluated in this article, or claim that may be made by its manufacturer, is not guaranteed or endorsed by the publisher.

## Supplementary material

Supplementary Figure 1 Expression analysis and survival analysis for *SFXN3* in pan-cancer by UALCAN and GEPIA. **(A)** The expression of *SFXN3* in pan-cancer based on TCGA cancer and normal data analyzed using UALCAN. **(B)** High expression of *SFXN3* in TCGA CHOL, HNSC, KIRC, LAML, PAAD, PCPG, and SARC tissues compared with corresponding TCGA and GTEx normal tissues, as analyzed using the GEPIA database. **(C, D)** The overall survival (OS) analysis and disease-free survival (DFS) analysis in patients with HNSC determined using the GEPIA database. **p* value < 0.05; ***p* value < 0.01; ****p* value < 0.001. Transcripts per million (TPM) values were employed for the generation of boxplots and were used to estimate the significance of the difference in gene expression levels between the groups. The *t* test was performed using a PERL script with the Comprehensive Perl Archive Network (CPAN) module in the GEPIA database.

Supplementary Figure 2 Tumor-infiltrating immune cells in HNSC samples were estimated by xCell. **p* value < 0.05; ***p* value < 0.01; ****p* value < 0.001

Supplementary Figure 3 Drug activity of commonly used chemotherapeutics between high and low *SFXN3* expression groups. Activity z scores of chemotherapeutics, including paclitaxel **(A)**, docetaxel **(B)**, methotrexate **(C)**, carboplatin **(D)**, fluorouracil **(E)**, cisplatin **(F)**, 5-fluoro deoxy uridine 10mer **(G)**, and gemcitabine **(H)** in the high *SFXN3* expression group compared with those in the low *SFXN3* expression group. Patients with high *SFXN3* expression were found to possess lower activity z scores for FDA-approved chemotherapeutics such as docetaxel and paclitaxel. The *p*-values were calculated using the Wilcoxon test. NS, not significant; **p* value < 0.05; ***p* value < 0.01.

Supplementary Figure 4
**(A)** Expression analysis of *SFXN1* in pan-cancer. **(B)** Analysis of overall survival (OS) analysis and disease-free survival (DFS) related to *SFXN1* expression in HNSC.

Supplementary Table 1 The expression of SFNX3 and IC50 values of common drugs in different HNSC cell lines.

Supplementary Table 2 Predicted miRNAs by eight prediction programs.
