# Supplementary figures and images for: Non-coding RNA-mediated high expression of SFXN3 as a prognostic biomarker associated with paclitaxel resistance and immunosuppressive microenvironment in head and neck cancer
Source: Front Immunol. 2022 Sep 8;13:920136. doi: 10.3389/fimmu.2022.920136 (PMC9493355; doi:10.3389/fimmu.2022.920136)

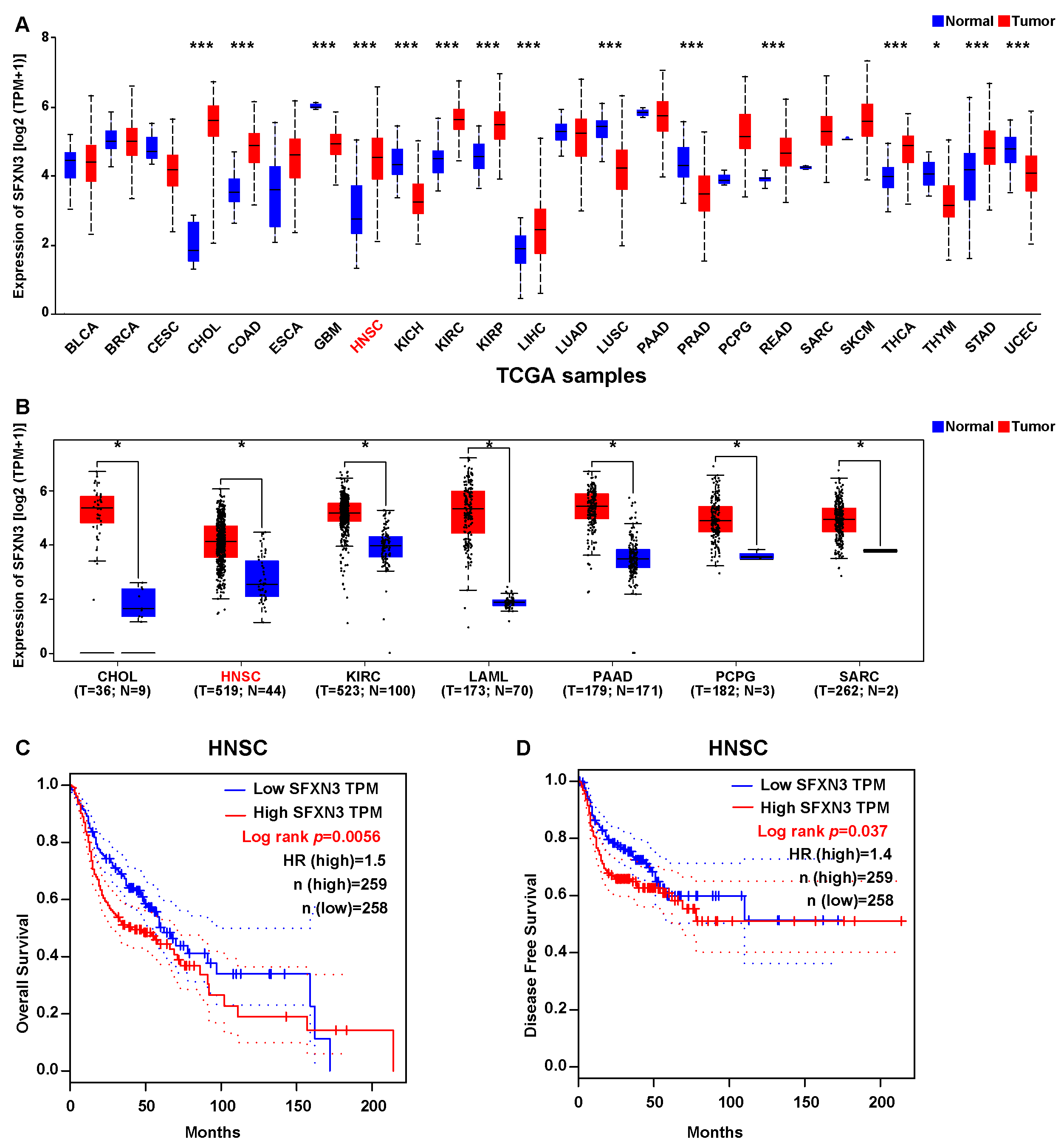

Supplement: Supplementary Figure 1 — Expression analysis and survival analysis for SFXN3 in pan-cancer by UALCAN and GEPIA. (A) The expression of SFXN3 in pan-cancer based on TCGA cancer and normal data analyzed using UALCAN. (B) High expression of SFXN3 in TCGA CHOL, HNSC, KIRC, LAML, PAAD, PCPG, and SARC tissues compared with corresponding TCGA and GTEx normal tissues, as analyzed using the GEPIA database. (C, D) The overall survival (OS) analysis and disease-free survival (DFS) analysis in patients with HNSC determined using the GEPIA database. *p value < 0.05; **p value < 0.01; ***p value < 0.001. Transcripts per million (TPM) values were employed for the generation of boxplots and were used to estimate the significance of the difference in gene expression levels between the groups. The t test was performed using a PERL script with the Comprehensive Perl Archive Network (CPAN) module in the GEPIA database. [file Image_1.tif]

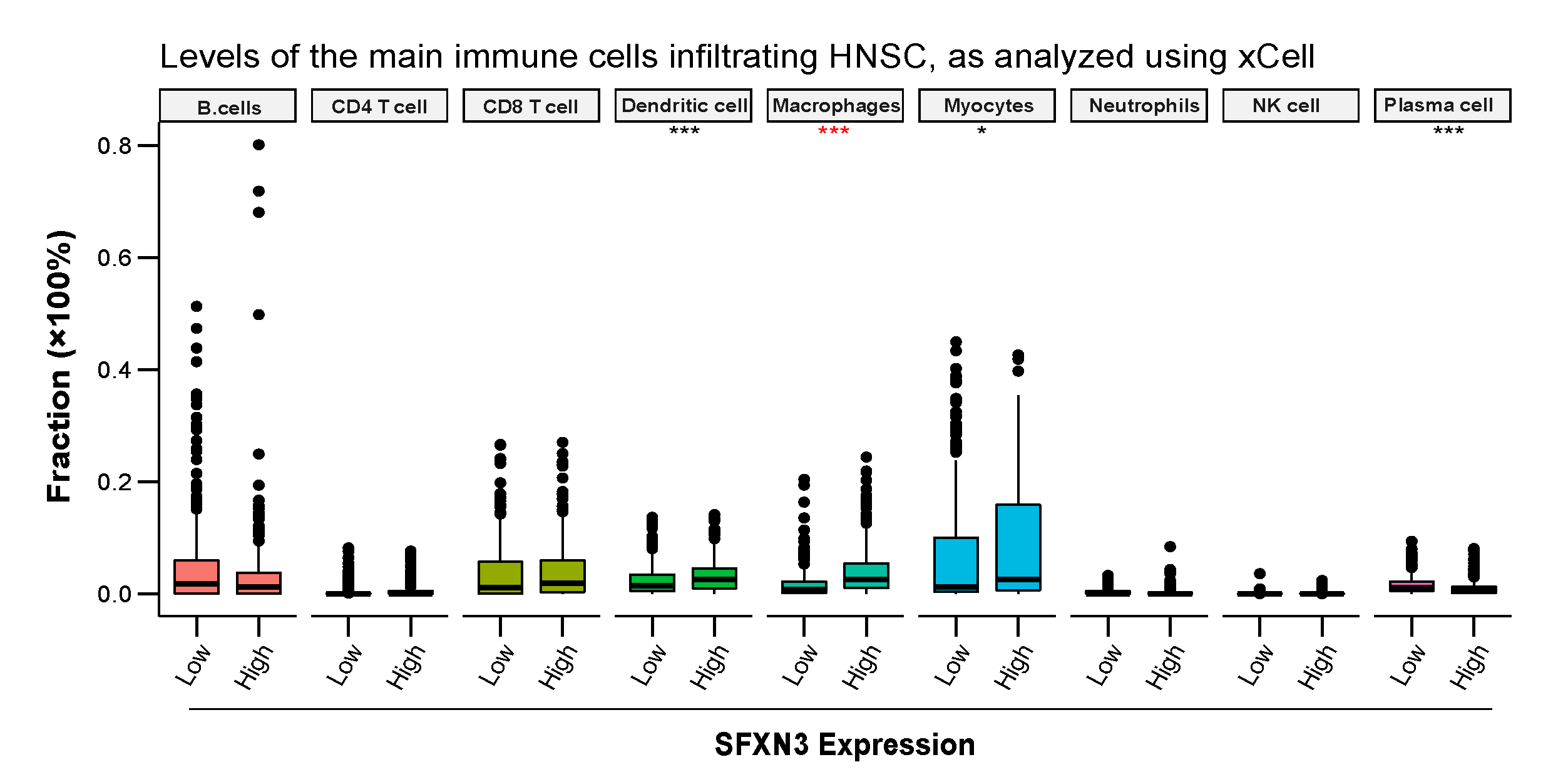

Supplement: Supplementary Figure 2 — Tumor-infiltrating immune cells in HNSC samples were estimated by xCell. *p value < 0.05; **p value < 0.01; ***p value < 0.001 [file Image_2.tif]

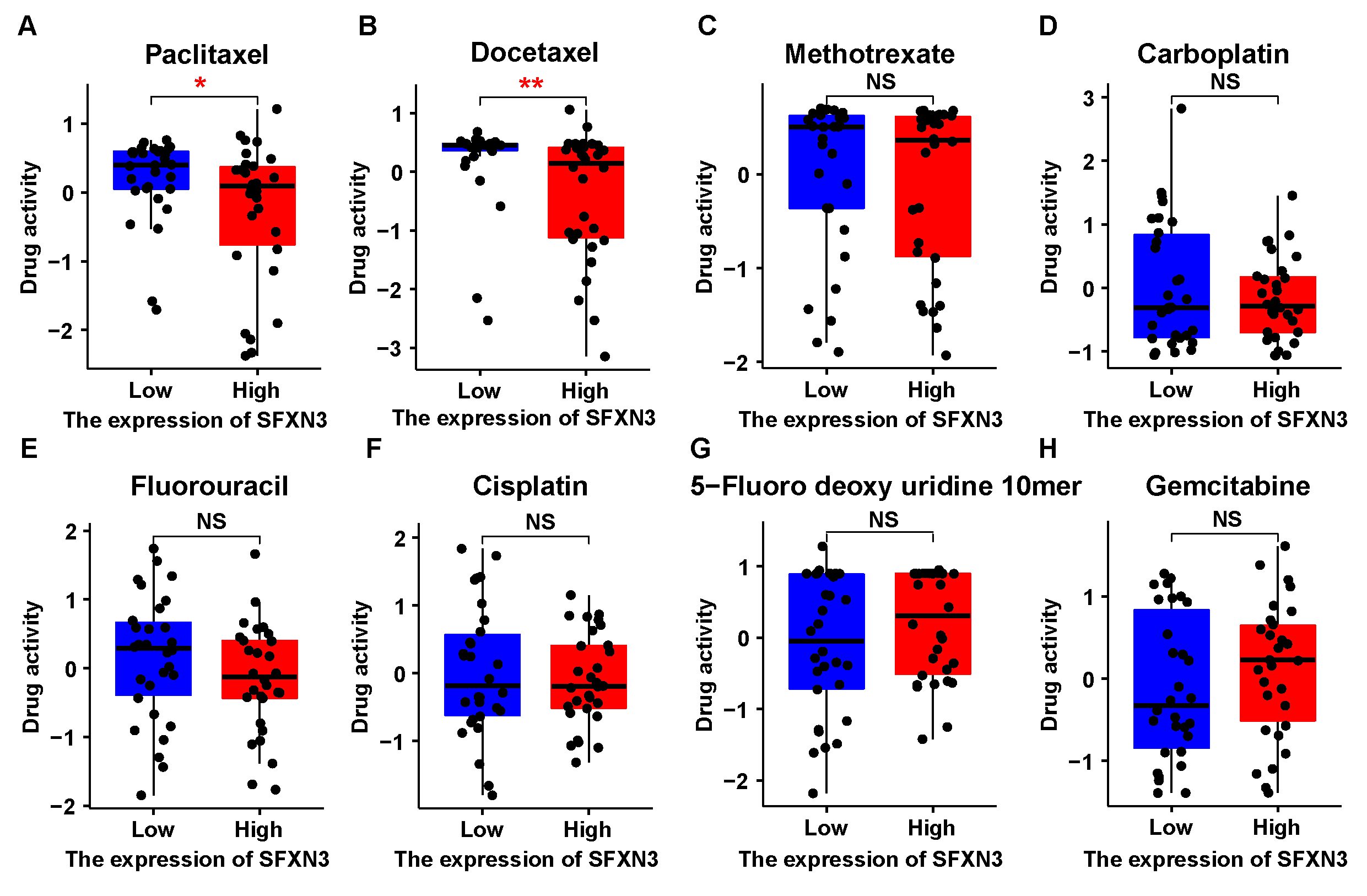

Supplement: Supplementary Figure 3 — Drug activity of commonly used chemotherapeutics between high and low SFXN3 expression groups. Activity z scores of chemotherapeutics, including paclitaxel (A), docetaxel (B), methotrexate (C), carboplatin (D), fluorouracil (E), cisplatin (F), 5-fluoro deoxy uridine 10mer (G), and gemcitabine (H) in the high SFXN3 expression group compared with those in the low SFXN3 expression group. Patients with high SFXN3 expression were found to possess lower activity z scores for FDA-approved chemotherapeutics such as docetaxel and paclitaxel. The p-values were calculated using the Wilcoxon test. NS, not significant; *p value < 0.05; **p value < 0.01. [file Image_3.tif]

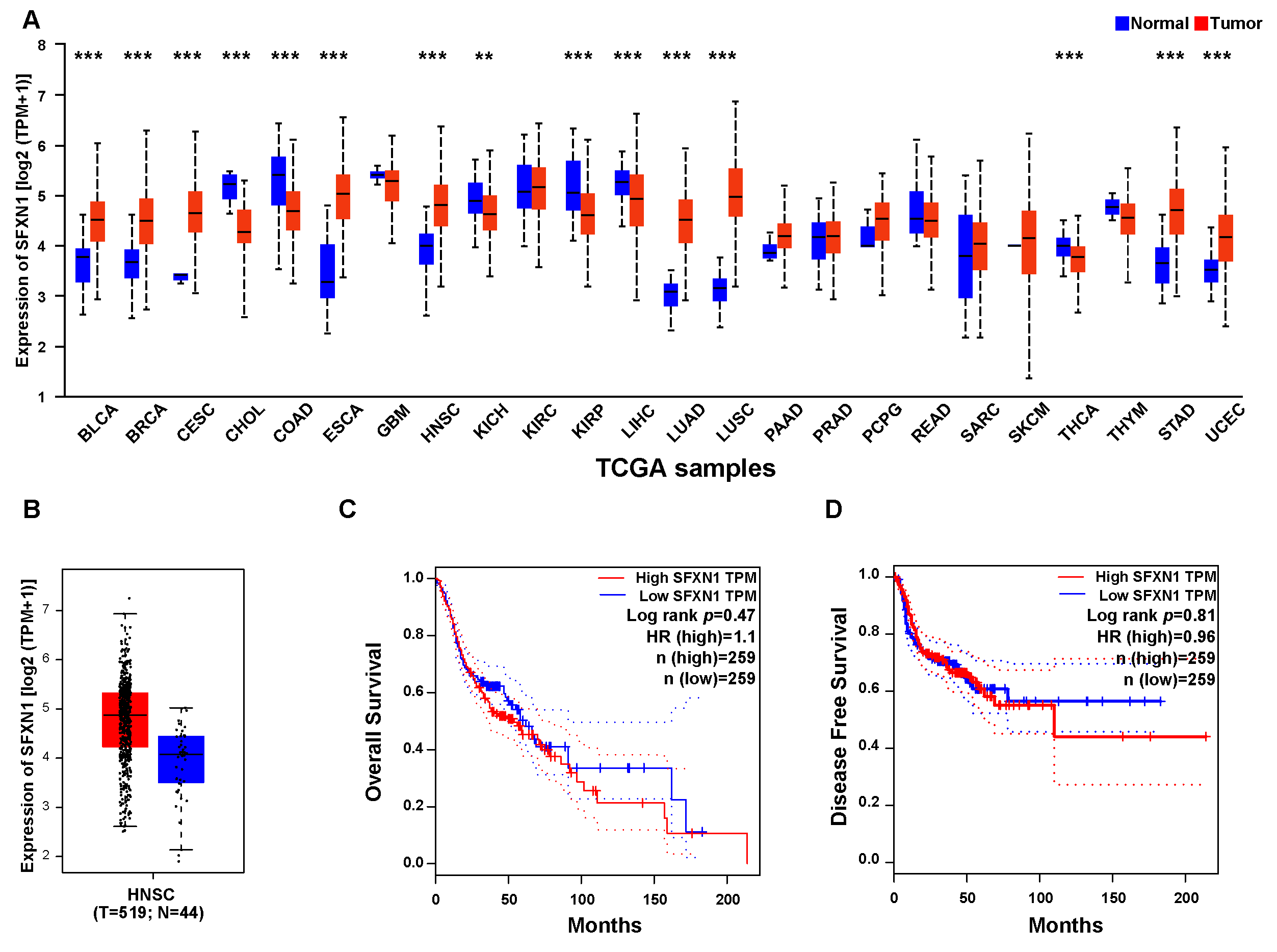

Supplement: Supplementary Figure 4 — (A) Expression analysis of SFXN1 in pan-cancer. (B) Analysis of overall survival (OS) analysis and disease-free survival (DFS) related to SFXN1 expression in HNSC. [file Image_4.tif]
